# Monepantel is a non-competitive antagonist of nicotinic acetylcholine receptors from *Ascaris suum* and *Oesophagostomum dentatum*

**DOI:** 10.1016/j.ijpddr.2017.12.001

**Published:** 2017-12-16

**Authors:** Melanie Abongwa, Djordje S. Marjanovic, James G. Tipton, Fudan Zheng, Richard J. Martin, Sasa M. Trailovic, Alan P. Robertson

**Affiliations:** a Department of Biomedical Sciences, College of Veterinary Medicine, Iowa State University, Ames, IA 50011, USA; b Department of Pharmacology and Toxicology, College of Veterinary Medicine, University of Belgrade, Belgrade, Serbia; c Department of Chemistry, College of Liberal Arts and Sciences, Iowa State University, Ames, IA 50011, USA

**Keywords:** Monepantel, Zolvix^®^, Nicotinic acetylcholine receptors, Mode of action, Non-competitive antagonist, mptl, monepantel, ach, acetylcholine, nAChR, nicotinic acetylcholine receptor, STH, Soil-Transmitted Helminth, *Asu*, *Ascaris suum*, *Ode*, *Oesophagustomum dentatum*, *Hco*, *Haemonchus contortus*, GI, gastro-intestinal, AADs, amino-acetonitrile derivatives, LGIC, ligand-gated ion channel, APF, *Ascaris* Perienteric Fluid, DMSO, dimethyl sulfoxide

## Abstract

Zolvix^®^ is a recently introduced anthelmintic drench containing monepantel as the active ingredient. Monepantel is a positive allosteric modulator of DEG-3/DES-2 type nicotinic acetylcholine receptors (nAChRs) in several nematode species. The drug has been reported to produce hypercontraction of *Caenorhabditis elegans* and *Haemonchus contortus* somatic muscle. We investigated the effects of monepantel on nAChRs from *Ascaris suum* and *Oesophagostomum dentatum* heterologously expressed in *Xenopus laevis* oocytes. Using two-electrode voltage-clamp electrophysiology, we studied the effects of monepantel on a nicotine preferring homomeric nAChR subtype from *A. suum* comprising of ACR-16; a pyrantel/tribendimidine preferring heteromeric subtype from *O. dentatum* comprising UNC-29, UNC-38 and UNC-63 subunits; and a levamisole preferring subtype (*O. dentatum*) comprising UNC-29, UNC-38, UNC-63 and ACR-8 subunits. For each subtype tested, monepantel applied in isolation produced no measurable currents thereby ruling out an agonist action. When monepantel was continuously applied, it reduced the amplitude of acetylcholine induced currents in a concentration-dependent manner. In all three subtypes, monepantel acted as a non-competitive antagonist on the expressed receptors. ACR-16 from *A. suum* was particularly sensitive to monepantel inhibition (*IC*_*50*_ values: 1.6 ± 3.1 nM and 0.2 ± 2.3 μM). We also investigated the effects of monepantel on muscle flaps isolated from adult *A. suum*. The drug did not significantly increase baseline tension when applied on its own. As with acetylcholine induced currents in the heterologously expressed receptors, contractions induced by acetylcholine were antagonized by monepantel. Further investigation revealed that the inhibition was a mixture of competitive and non-competitive antagonism. Our findings suggest that monepantel is active on multiple nAChR subtypes.

## Highlights

• Monepantel is a positive allosteric modulator of nematode DEG-3/DES-2 type nAChRs.
• We found monepantel inhibited acetylcholine induced contractions in *Ascaris suum.*
• Monepantel is a non-competitive antagonist of the *Ascaris* ACR-16 (nicotine preferring) nAChR.
• Monepantel inhibits *O. dentatum* levamisole & pyrantel sensitive nAChRs non-competitively.
• The mode of action of monepantel is complex and likely involves multiple targets.

## Graphical abstract

**Figure:**
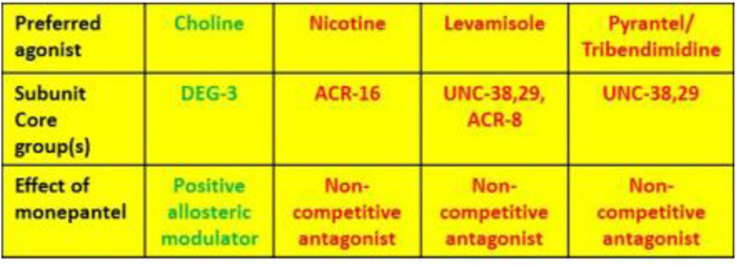

## 1 Introduction

Soil-Transmitted Helminth (STH) infections in humans and animals cause significant disease (morbidity & mortality) globally. At least 1.2 billion people suffer from STH infections, with an estimated at-risk population of 4.5 billion (Bethony et al., 2006, Brooker et al., 2006, Lammie et al., 2006). Control of these infections is heavily reliant on the use of anthelmintics as there are no effective vaccines and sanitation is often sub-optimal (Kaminsky et al., 2013). There are a limited number of drugs used to treat helminth infections (Keiser and Utzinger, 2008). Antinematodal (anthelmintic) drugs can be classified on the basis of similarity in chemical structure; benzimidazoles, imidazothiazoles, tetrahydopyrimidines, macrocyclic lactones, amino-acetonitrile derivatives, spiroindoles and cyclooctadepsipeptides. The benzimidazoles, imidazothiazoles, tetrahydopyrimidines and macrocyclic lactones are older anthelmintic drug classes. Increasing reports of resistance to the ‘older’ anthelmintic drugs has encouraged the development of newer drug classes: amino-acetonitrile derivatives, spiroindoles and cyclooctadepsipeptides.

**Fig. 1:**
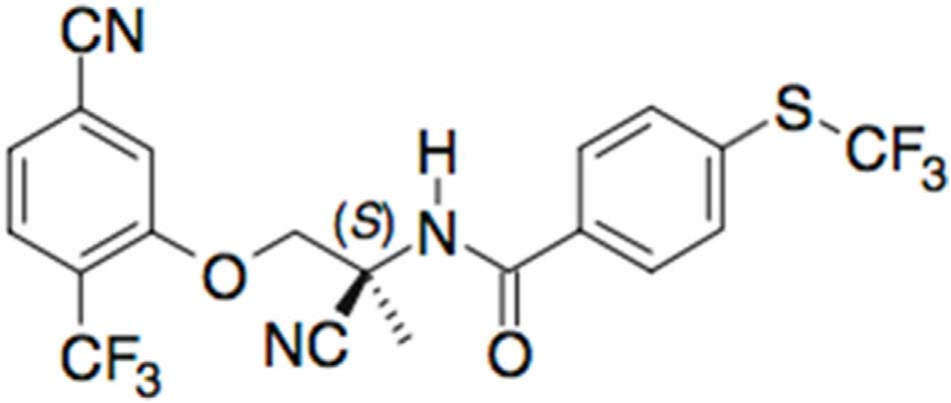
Chemical structure of monepantel [N-[(2S)-2-cyano-1-[5-cyano-2-(trifluoromethyl)phenoxy]propan-2-yl]-4-(trifluoromethylsulfanyl)benzamide].

Zolvix^®^ (Novartis Animal Health, Greensboro, NC, USA) is a recently developed anthelmintic for control of gastro-intestinal (GI) nematodes in sheep. It was first introduced in New Zealand in 2009, and contains 25 mg/ml monepantel (mptl) as the active ingredient (Fig. 1). Monepantel is the first member of the amino-acetonitrile derivative (AAD) group of anthelmintics. It has a wide range of activity against nematodes in sheep, including those resistant to benzimidazoles, imidazothiazoles and macrocyclic lactones (Ducray et al., 2008, Kaminsky et al., 2008a, Kaminsky et al., 2008b). Disappointingly, resistance has developed in infected goats and sheep following treatment with monepantel. The first field report of monepantel resistance was observed in *Teladorsagia circumcincta* and *Trichostrongylus colubriformis* in goats and sheep on a farm in the lower North Island in New Zealand <2 years after its initial use on that farm (Scott et al., 2013). Subsequent cases of resistance to monepantel have been reported for *H. contortus* in sheep (Mederos et al., 2014, Van den Brom et al., 2015). The emergence of field resistance to monepantel within very short periods following treatment underscores the pressing need to understand the full mode of action of the drug and thus possible mechanisms of resistance.

Monepantel has a site of action reported to involve ACR-23, a member of the DEG-3/DES-2 group of nicotinic acetylcholine receptors (nAChRs) (Lecova et al., 2014). Kaminsky et al. (2008a) showed AADs to cause hypercontraction of body wall muscles in *Caenorhabditis elegans* and *Haemonchus contortus*, leading to spastic paralysis and subsequent death of the worms. These authors further revealed *C. elegans* treated with AADs display molting deficits and characteristics of necrosis (Kaminsky et al., 2008a). Subsequent mutagenesis screens in *H. contortus* led to the identification of the nAChR subunit gene, *mptl-1* (*acr-23*), as a target for AADs (Rufener et al., 2009). Comparative genomics of all ligand-gated ion channel (LGIC) genes from different clades of nematodes reported nematode species lacking the ACR-23/MPTL-1 nAChR subunits to be insensitive to monepantel. On the contrary, nematode species having ACR-23/MPTL-1 were susceptible to monepantel, thus promoting ACR-23/MPTL-1 nAChR as a principal target for the AADs (Rufener et al., 2010b). To further elucidate the mode of action of monepantel, Rufener et al. (2010b) showed that monepantel on its own did not activate heterologously expressed *H. contortus* DEG-3/DES-2 receptors but acted as a type 2 positive allosteric modulator when co-applied with choline (Rufener et al., 2010a). In heterologously expressed *C. elegans* ACR-23 receptors, monepantel caused potentiation following activation by betaine (Peden et al., 2013). Monepantel also acted as a positive allosteric modulator of *H. contortus* MPTL-1 and *C. elegans* ACR-20 receptors at low concentrations (<1 nM) but as a direct agonist at high concentrations (>0.1 μM) (Baur et al., 2015).

Interestingly, a homolog of *C. elegans acr-23* is present in the *A. suum* genome (Jex et al., 2011). However, *A. suum* is not susceptible to monepantel treatment *in vivo* (Tritten et al., 2011). Our research seeks to advance knowledge on the mode of action of monepantel on nAChRs from Clade III (*A. suum*) and Clade V (*Oesophagostomum dentatum*) nematodes. This present study therefore is an investigation of the effects of monepantel on nAChRs that are widely and ubiquitously expressed in *A. suum* (*Asu-*ACR-16), and those involved in neurotransmission (pyrantel/tribendimidine sensitive and levamisole sensitive nAChRs) in *O. dentatum*. We find that monepantel also acts selectively as an antagonist on the nematode nAChRs we studied.

## 2 Materials and methods

### 2.1 Xenopus oocyte expression

All nAChR subunit and ancillary factor cRNAs from *A. suum* (*Asu-acr-16* and *Asu-ric-3*), *O. dentatum* (*Ode-unc-29*, *Ode-unc-38*, *Ode-unc-68* and *Ode-acr-8*) and *H. contortus* (*Hco-ric-3*, *Hco-unc-50* and *Hco-unc-74*) were prepared as previously described (Abongwa et al., 2016a, Buxton et al., 2014). Briefly, defolliculated *X. laevis* oocytes were obtained from Ecocyte Bioscience (Austin, TX, USA). A Nanoject II microinjector (Drummond Scientific, Broomall, PA, USA) was used to inject cRNA into the cytoplasm at the animal pole region of the oocytes. Homomeric nAChRs comprising *Asu-*ACR-16 subunits were expressed by co-injecting 25 ng of *Asu*-*acr-16* with 5 ng of *Asu-ric-3* in a total volume of 50 nl in nuclease-free water. Heteromeric nAChRs from *O. dentatum* were expressed by co-injecting 1.8 ng of each subunit cRNA that make up the levamisole (*Ode-unc-29*, *Ode-unc-38*, *Ode-unc-68* and *Ode-acr-8*) or pyrantel/tribendimidine (*Ode-unc-29*, *Ode-unc-38* and *Ode-unc-63*) receptor with 1.8 ng of each *H. contortus* ancillary factor (*Hco-ric-3*, *Hco-unc-50* and *Hco-unc-74*) in a total volume of 36 nl in nuclease-free water. Injected oocytes were transferred to 96-well microtiter plates containing 200 μl incubation solution (100 mM NaCl, 2 mM KCl, 1.8 mM CaCl_2_·2H_2_0, 1 mM MgCl_2_·6H_2_0, 5 mM HEPES, 2.5 mM Na pyruvate, 100 U·ml^−1^penicillin and 100 μg ml^−1^ streptomycin, pH 7.5) and incubated at 19 °C for 5–7 days to allow for functional receptor expression. Incubation solution was changed daily.

### 2.2 Two-electrode voltage-clamp electrophysiology

Currents produced by activation of expressed *Asu-*ACR-16 and *Ode* levamisole sensitive and *Ode* pyrantel/tribendimidine sensitive receptors were recorded using the two-electrode voltage-clamp electrophysiology technique as previously described (Abongwa et al., 2016a, Buxton et al., 2014). 100 μM BAPTA-AM was added to the oocyte incubation solution about 4 h prior to recording to prevent activation of endogenous calcium-activated chloride currents during recording. Recordings were made using an Axoclamp 2B amplifier (Molecular Devices, Sunnyvale, CA, USA) and data acquired on a computer with Clampex 10.3 (Molecular Devices, Sunnyvale, CA, USA). For all experiments, oocytes were voltage-clamped at −60 mV. Microelectrodes for impaling oocytes were pulled using a Flaming/Brown horizontal electrode puller (Model P-97; Sutter Instruments, Novato, CA, USA). The microelectrodes were filled with 3 M KCl and their tips carefully broken with a piece of tissue paper to attain a resistance of 2–5 MΩ in recording solution (100 mM NaCl, 2.5 mM KCl, 1 mM CaCl_2_·2H_2_O and 5 mM HEPES, pH 7.3).

### 2.3 Muscle contraction assay

Adult *A. suum* were collected from the IMES slaughterhouse, Belgrade, Serbia and maintained in Locke's solution (155 mM NaCl, 5 mM KCl, 2 mM CaCl_2_, 1.5 mM NaHCO_3_ and 5 mM glucose) at 32 °C for 5 days. Locke's solution was changed daily. *Ascaris* muscle flaps for contraction studies were prepared as described in Trailović et al. (2015). Briefly, 1 cm muscle body flaps were prepared by dissecting the anterior 2–3 cm part of the worm. A force transducer in an experimental bath containing 20 ml APF (23 mM NaCl, 110 mM Na acetate, 24 mM KCl, 6 mM CaCl_2_, 5 mM MgCl_2_, 11 mM glucose, 5 mM HEPES, pH 7.6) and 0.1% DMSO and bubbled with nitrogen was attached to each muscle flap. The bath was maintained at 37 °C during which isometric contractions of each flap were monitored on a PC computer using a BioSmart interface and eLAB software (EIUnit, Belgrade). The system allows real time recording, display and analysis of experimental data. The preparation was allowed to equilibrate for 15 min under an initial tension of 0.5 g after which acetylcholine (1–100 μM) in the absence and presence of monepantel (1–30 μM) was applied to the preparation.

### 2.4 Drugs

Acetylcholine was purchase from Sigma-Aldrich (St Louis, MO, USA). Zolvix (monepantel 25 mg/ml) was a gift from Dr Michael Kimber (Iowa State University, Ames, IA). Acetylcholine was dissolved in either APF or oocyte recording solution. Monepantel was dissolved in DMSO such that the final DMSO concentration did not exceed 0.1%. All other chemicals were purchased from Sigma-Aldrich (St Louis, MO, USA) or Fisher Scientific (Hampton, NH, USA).

### 2.5 Data analysis

#### 2.5.1 Electrophysiology

Electrophysiology data was measured with Clampfit 10.3 (Molecular devices, Sunnyvale CA, USA) and analyzed with GraphPad Prism 5.0 (GraphPad Software Inc., La Jolla, CA, USA). All concentration-response experiments began with a 10 s application of 100 μM acetylcholine. Increasing concentrations of acetylcholine (0.1–100 μM) were applied for 10 s at ∼2 min intervals. For experiments using monepantel, an initial 10 s application of 100 μM acetylcholine was followed after 2 min by continuous perfusion of a single concentration of monepantel (0.3 nM - 30 μM); for the rest of the experiment 10 s applications of acetylcholine (0.3–100 μM) were used at ∼2 min intervals in the presence of monepantel. Responses to each acetylcholine concentration were normalized to the initial control 100 μM acetylcholine responses and expressed as mean ± s.e.m. Concentration-response relationships (for each oocyte) were analyzed by fitting log concentration-response data points with the Hill equation as previously described (Boulin et al., 2008). % inhibition for monepantel was calculated using 100-*R*_*max*_ for each experiment and plotted as mean ± s.e.m. on the concentration-inhibition plots. *IC*_*50*_'s were calculated as previously described (Zheng et al., 2016). We used one-way ANOVA to test for statistical differences among treatment groups. If the group means were statistically different (*p* < .05), we used the Tukey multiple comparison test to determine significant differences between groups.

#### 2.5.2 Muscle contraction

Isometric contractions of each *Ascaris* muscle flap in the absence and presence of monepantel were monitored on a PC computer using eLAB software (EIUnit, Belgrade). GraphPad Prism 5.0 (GraphPad Software Inc., La Jolla, CA, USA) was used to analyze the data. Responses to each acetylcholine concentration in the absence and presence of monepantel were expressed as mean ± s.e.m. Single concentration-response relationships were fitted to the data as described in Section 2.5.1.

## 3 Results

### 3.1 Effects of monepantel on Asu-ACR-16

**Fig. 2:**
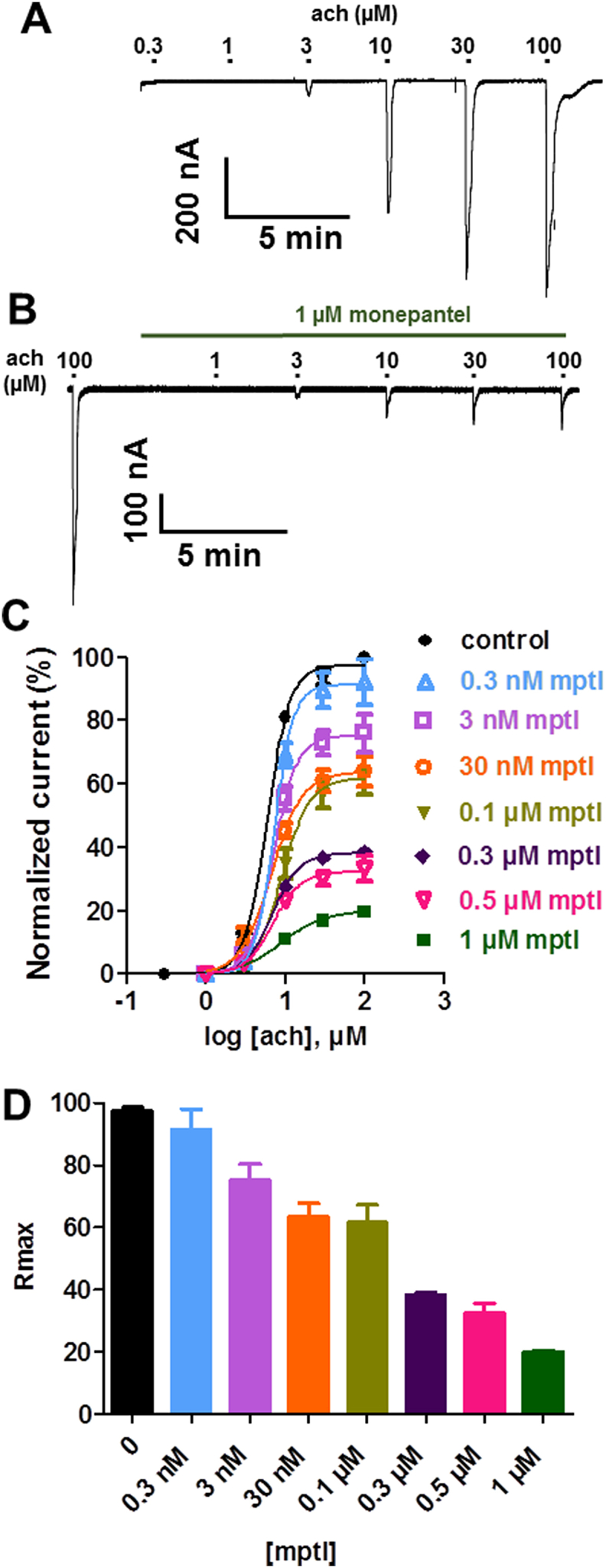
Inhibitory effects of monepantel on *Asu-*ACR-16 (nicotine sensitive receptors). (A) Sample traces for two-electrode voltage-clamp recording for oocyte responses to acetylcholine for oocytes expressing *Asu-*ACR-16 subtype nAChR. (B) Sample traces for two-electrode voltage-clamp recording for oocyte responses to acetylcholine in the presence of 1 μM monepantel. (C) Concentration-response plots for acetylcholine in the absence and presence of monepantel. Control acetylcholine (n = 4, black); in the presence of 0.3 nM monepantel (n = 4, light blue); 3 nM monepantel (n = 4, light purple); 30 nM monepantel (n = 4, orange), 0.1 μM monepantel (n = 5, olive green), 0.3 μM monepantel (n = 4, purple), 0.5 μM monepantel (n = 4, pink) and 1 μM monepantel (n = 4, green). (D) Bar chart showing mean ± s.e.m. (n = 4–5) for *R*_max_ for acetylcholine and monepantel. (For interpretation of the references to colour in this figure legend, the reader is referred to the Web version of this article.)

*Asu*-ACR-16 is a homomeric nAChR comprising of *Asu-*ACR-16 subunits (Abongwa et al., 2016a). This receptor subtype requires only the ancillary factor *Asu-*RIC-3 for functional expression in *Xenopus* oocytes. ACR-16 is nicotine sensitive, but levamisole insensitive, and is commonly referred to as the nicotine subtype nAChR (Abongwa et al., 2016a, Ballivet et al., 1996, Raymond et al., 2000, Sattelle et al., 2009). We tested the effects of different monepantel concentrations (0.3 nM - 1 μM) on *Asu*-ACR-16 responses to acetylcholine. For control experiments, each acetylcholine concentration from 0.3 to 100 μM was applied for 10s, Fig. 2A. To test the effects of monepantel, 100 μM acetylcholine was first applied for 10s as control, followed by a 2 min wash, then perfusion with monepantel, after which acetylcholine applications were repeated in the presence of monepantel, Fig. 2B. For both control and test experiments, a 2 min wash period was applied between drug applications. Monepantel applied on its own did not cause activation of *Asu*-ACR-16, eliminating agonist actions on this receptor subtype. When co-applied with acetylcholine, monepantel caused a concentration-dependent inhibition of *Asu*-ACR-16 responses to acetylcholine. Fig. 2C shows concentration-response plots for these experiments. Monepantel did not change the *EC*_*50*_ but did significantly reduce *R*_max_, Fig. 2C and D, implying non-competitive antagonism. *EC*_*50*_ and *R*_max_ values for these observations are shown in Table 1.

**Table 1:** Effect of monepantel on acetylcholine *EC*_*50*_ and *R*_max_ values for *Asu-*ACR-16.

	*EC*_*50*_ (mean ± s.e.m., n)	*R*_max_ (mean ± s.e.m., n)
Acetylcholine	5.9 ± 0.3 μM, n = 4	97.5 ± 1.2%, n = 4
+0.3 nM monepantel	7.3 ± 0.0 μM, n = 4	91.4 ± 6.6%, n = 4
+3 nM monepantel	7.1 ± 0.1 μM, n = 4	75.3 ± 5.1%, n = 4
+30 nM monepantel	6.8 ± 0.8 μM, n = 4	63.6 ± 4.2%, n = 4
+0.1 μM monepantel	9.2 ± 1.0 μM, n = 5	61.6 ± 5.8%, n = 5
+0.3 μM monepantel	6.9 ± 0.2 μM, n = 4	38.2 ± 0.8%, n = 4
+0.5 μM monepantel	7.0 ± 0.5 μM, n = 4	32.5 ± 3.2%, n = 4
+1 μM monepantel	9.1 ± 1.3 μM, n = 4	19.9 ± 0.5%, n = 4

### 3.2 Effects of monepantel on expressed Ode pyrantel/tribendimidine receptors

**Fig. 3:**
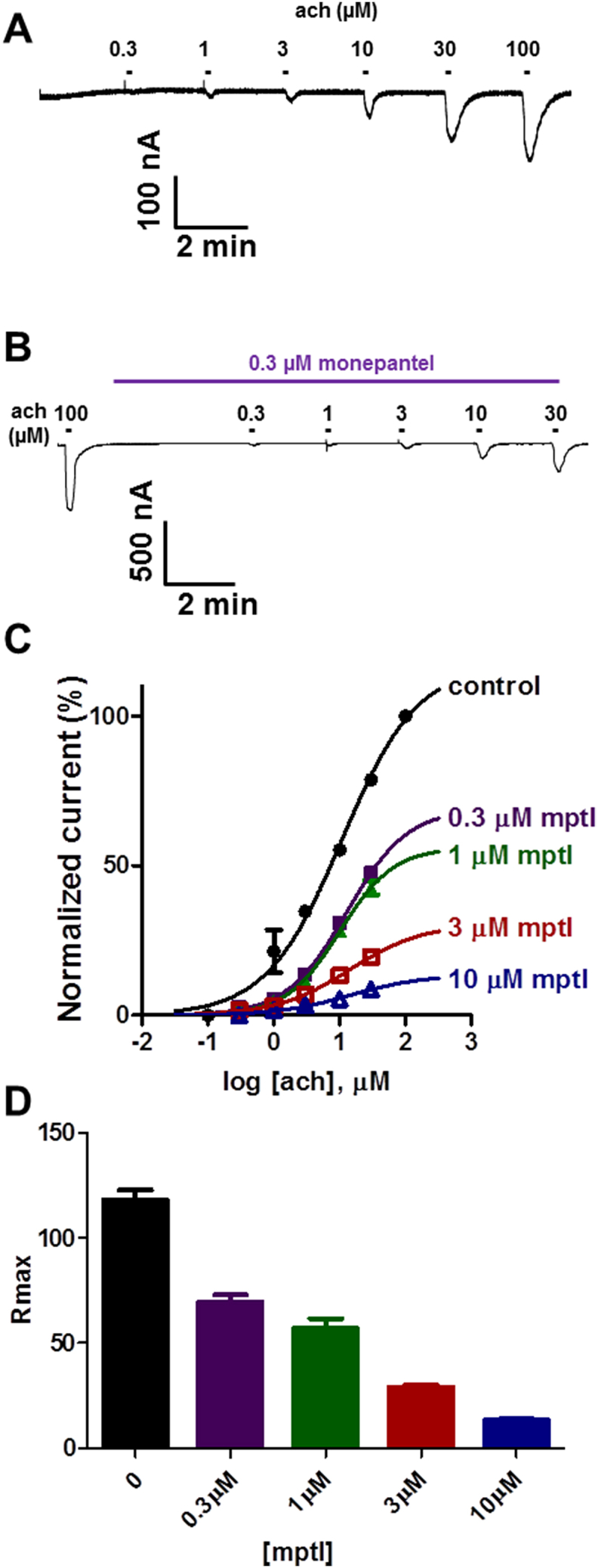
Inhibitory effects of monepantel on *Ode* pyrantel/tribendimidine preferring nAChRs. (A) Sample traces for two-electrode voltage-clamp recording for oocyte responses to acetylcholine for oocytes expressing *Ode* pyrantel/tribendimidine subtype nAChR. (B) Sample traces for two-electrode voltage-clamp recording for oocyte responses to acetylcholine in the presence of 0.3 μM monepantel. (C) Concentration-response plots for acetylcholine in the absence and presence of monepantel. Control acetylcholine (n = 4, black); in the presence of 0.3 μM monepantel (n = 5, purple); 1 μM monepantel (n = 4, green); 3 μM monepantel (n = 4, red) and 10 μM monepantel (n = 4, blue). (D) Bar chart showing mean ± s.e.m. (n = 4–5) for *R*_max_ for acetylcholine and monepantel. (For interpretation of the references to colour in this figure legend, the reader is referred to the Web version of this article.)

*Ode* pyrantel/tribendimidine receptors were described by Buxton et al. (2014) to comprise of the nAChR subunits *Ode-*UNC-29, *Ode-*UNC-38 and *Ode-*UNC-63. *Ode* pyrantel/tribendimidine receptors require all 3 ancillary factors *Hco-*RIC-3, *Hco-*UNC-50 and *Hco-*UNC-74 from *H. contortus* for functional expression in oocytes. To investigate the effects of monepantel on expressed *Ode* pyrantel/tribendimidine receptors, we used the same experimental protocol described for *Asu-*ACR-16 receptors in Section 3.1. Fig. 3C shows concentration-response plots for these experiments. Monepantel alone did not activate *Ode* pyrantel/tribendimidine receptors ruling out an agonist action. When co-applied with acetylcholine, features of non-competitive antagonism (no change in *EC*_*50*_, reduction in *R*_max_) were seen for *Ode* pyrantel/tribendimidine receptors, Fig. 3C and D. Table 2 shows *EC*_*50*_ and *R*_max_ values for these experiments.

**Table 2:** Effect of monepantel on acetylcholine *EC*_*50*_ and *R*_max_ values for *Ode* pyrantel/tribendimidine receptors.

	*EC*_*50*_ (mean ± s.e.m., n)	*R*_max_ (mean ± s.e.m., n)
Acetylcholine	11.3 ± 1.3 μM, n = 4	118.2 ± 4.5%, n = 4
+0.3 μM monepantel	13.2 ± 1.4 μM, n = 5	69.4 ± 3.5%, n = 5
+1 μM monepantel	10.4 ± 1.0 μM, n = 4	56.8 ± 4.8%, n = 4
+3 μM monepantel	12.9 ± 1.5 μM, n = 4	29.0 ± 0.9%, n = 4
+10 μM monepantel	14.5 ± 1.6 μM, n = 4	13.4 ± 0.6%, n = 4

### 3.3 Effects of monepantel on expressed Ode levamisole receptors

**Fig. 4:**
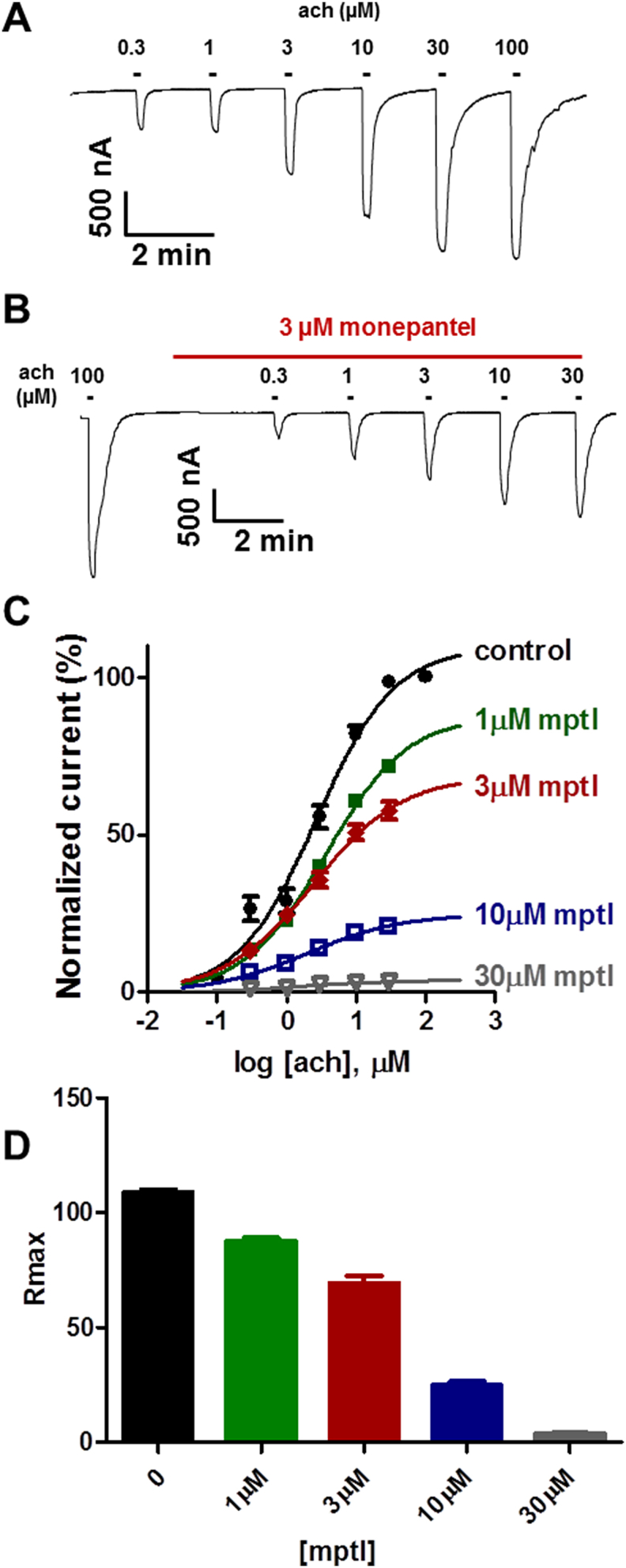
Monepantel inhibits *Ode* levamisole preferring receptors. (A) Sample traces for two-electrode voltage-clamp recording for oocyte responses to acetylcholine for oocytes expressing *Ode* levamisole subtype nAChR. (B) Sample traces for two-electrode voltage-clamp recording for oocyte responses to acetylcholine in the presence of 3 μM monepantel. (C) Concentration-response plots for acetylcholine in the absence and presence of monepantel. Control acetylcholine (n = 4, black); in the presence of 1 μM monepantel (n = 4, green); 3 μM monepantel (n = 4, red); 10 μM monepantel (n = 4, blue) and 30 μM monepantel (n = 5, grey). (D) Bar chart showing mean ± s.e.m. (n = 4–5) for *R*_max_ for acetylcholine and monepantel. (For interpretation of the references to colour in this figure legend, the reader is referred to the Web version of this article.)

Previous studies showed the nAChR subunits *Ode-*UNC-29, *Ode-*UNC-38, *Ode-*UNC-63 and *Ode-*ACR-8 are required to express the *Ode* levamisole sensitive receptors in *Xenopus* oocytes (Buxton et al., 2014). This was in accordance with previous work by Boulin et al. (2011) who expressed functional levamisole receptors in *Xenopus* oocytes when these oocytes were injected with *Hco-*UNC-29, *Hco-*UNC-38, *Hco-*UNC-63 and *Hco-*ACR-8 from *H. contortus*. The levamisole receptors required all 3 ancillary factors *Hco-*RIC-3, *Hco-*UNC-50 and *Hco-*UNC-74 from *H. contortus* for its functional expression in oocytes (Boulin et al., 2008, Boulin et al., 2011, Buxton et al., 2014). To investigate the effects of monepantel on expressed *Ode* levamisole receptors, we used the same experimental protocol described for *Asu-*ACR-16 receptors in Section 3.1. The monepantel concentrations tested on levamisole receptors were from 1 to 30 μM. Sample traces and concentration-response plots for these experiments are shown in Fig. 4A, B, & C. Again, monepantel applied alone failed to activate the *Ode* levamisole receptors, demonstrating no agonist action. As expected for a non-competitive antagonist, monepantel did not produce any significant change in *EC*_*50*_ but did cause a significant reduction in *R*_max_ as shown in Fig. 4C and D. *EC*_*50*_ and *R*_max_ values for these experiments are reported in Table 3.

**Table 3:** Effect of monepantel on acetylcholine *EC*_*50*_ and *R*_max_ values for *Ode* levamisole receptors.

	*EC*_*50*_ (mean ± s.e.m., n)	*R*_max_ (mean ± s.e.m., n)
Acetylcholine	2.7 ± 0.4 μM, n = 4	108.8 ± 1.0%, n = 4
+1 μM monepantel	3.8 ± 0.4 μM, n = 4	87.9 ± 1.2%, n = 4
+3 μM monepantel	2.7 ± 0.4 μM, n = 4	69.3 ± 3.2%, n = 4
+10 μM monepantel	2.2 ± 0.4 μM, n = 4	25.0 ± 1.4%, n = 4
+30 μM monepantel	1.8 ± 0.3 μM, n = 5	3.9 ± 0.1%, n = 5

### 3.4 Effects of monepantel on Ascaris body muscle flaps

**Fig. 5:**
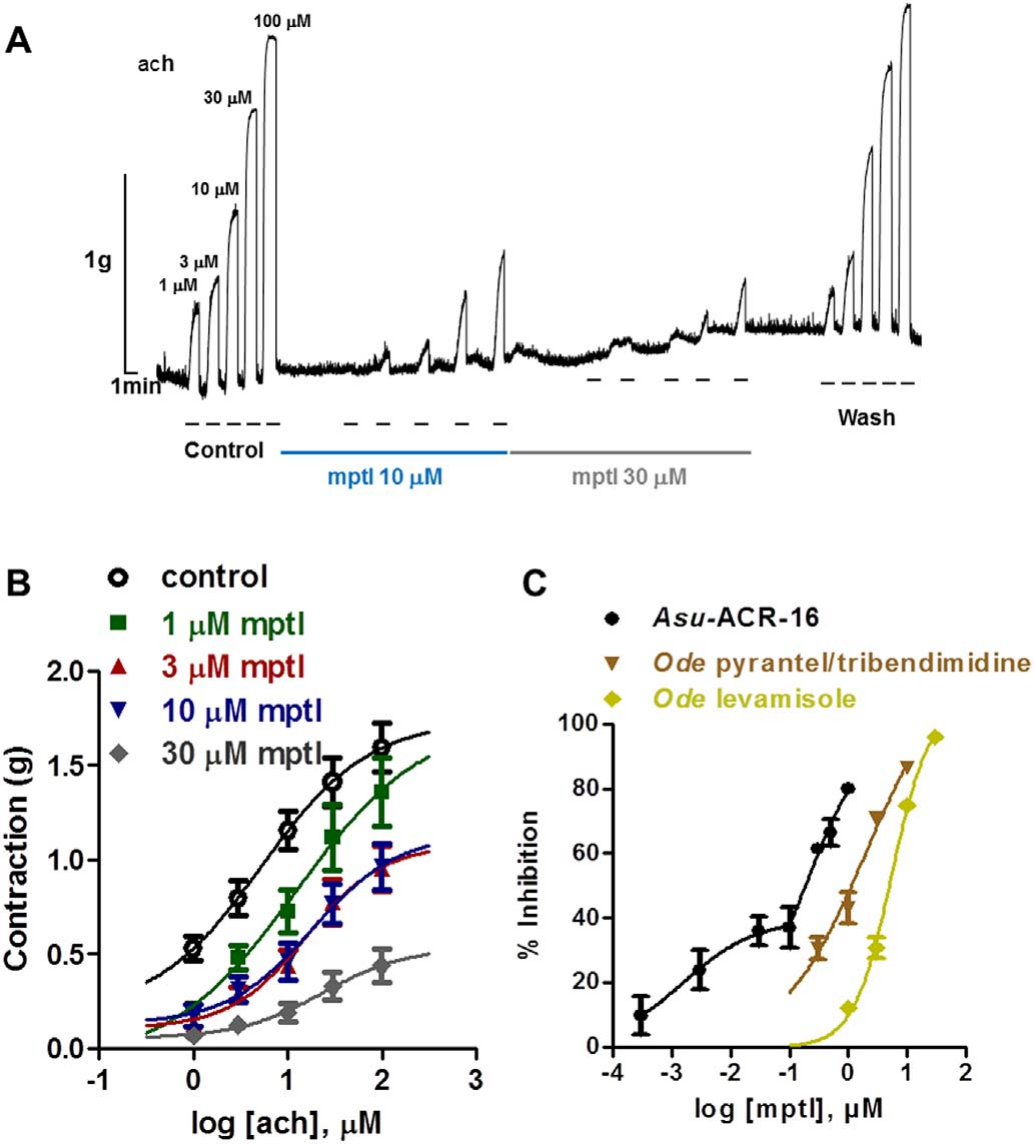
Inhibition of *Ascaris suum* muscle flap contractions by monepantel. (A) Isometric contractions of *A. suum* muscle flap following application of increasing acetylcholine concentrations and antagonism by 10 μM (blue bar) and 30 μM (grey bar) monepantel. (B) Concentration-contraction response plots for acetylcholine showing mean ± s.e.m. bars. Control acetylcholine (n = 11, black); in the presence of 1 μM monepantel (n = 6, green), 3 μM monepantel (n = 6, red), 10 μM monepantel (n = 5, blue) and 30 μM monepantel (n = 5, grey). (C) Inhibition curve plotted as mean ± s.e.m. (n = 4–5) maximum response versus concentration of monepantel and Hill equation fit with an *IC*_*50*_ of 1.6 ± 3.1 nM and 0.2 ± 2.3 μM for *Asu-*ACR-16, 1.7 ± 0.7 μM for *Ode* pyrantel/tribendimidine, and 5.0 ± 0.5 μM for *Ode* levamisole receptors. (For interpretation of the references to colour in this figure legend, the reader is referred to the Web version of this article.)

The results we obtained for expressed nicotine (*Asu-*ACR-16), *Ode* pyrantel/tribendimidine (*Ode*-UNC-29:*Ode*-UNC-38:*Ode*-UNC-63) and *Ode* levamisole (*Ode*-UNC-29:*Ode*-UNC-38:*Ode*-UNC-63:*Ode*-ACR-8) subtype nAChRs which we describe in Sections 3.1, 3.2, 3.3, encouraged us to investigate the *in vivo* effects of monepantel on *Ascaris* muscle. Fig. 5A shows representative traces of the effects of adding increasing concentrations of acetylcholine in the absence and presence of monepantel on isometric contractions of an *Ascaris* body flap preparation. Application of increasing concentrations of acetylcholine from 1 to 100 μM produced concentration-dependent contraction responses which were inhibited by monepantel in a concentration-dependent manner. Monepantel on its own did not produce any significant change in baseline tension. Washing reversed the inhibition caused by monepantel to near control levels. Concentration-response plots (mean ± s.e.m.) for acetylcholine and monepantel are shown in Fig. 5B. 1 μM monepantel produced a significant reduction in the maximum response, *R*_max_ and also shifted the *EC*_*50*_ to the right. Increasing the concentration of monepantel to 3, 10 and 30 μM further reduced the *R*_max_ and caused a further right-shift in the *EC*_*50*_, characteristic of a mixture of competitive and non-competitive antagonism. The activity of monepantel on *Ascaris* muscle flaps was rather interesting, as previous authors have showed monepantel (600 mg/kg) to lack *in vivo* activity against *A. suum* (Tritten et al., 2011). Table 4 shows *EC*_*50*_ and *R*_max_ values for acetylcholine alone and in the presence of 1–30 μM monepantel.

**Table 4:** Effect of monepantel on acetylcholine *EC*_*50*_ and *R*_max_ values for *A. suum* muscle flaps (n = 5–11 for each data point fitted).

	*EC*_*50*_ (log *EC*_*50*_ ± s.e.m.)	*R*_max_ (mean ± s.e.m.)
Acetylcholine	5.3 μM (0.7 ± 0.5)	1.7 ± 0.4 g
+1 μM monepantel	12.5 μM (1.1 ± 0.6)	1.8 ± 1.3 g
+3 μM monepantel	16.0 μM (1.2 ± 0.3)	1.1 ± 0.3 g
+10 μM monepantel	18.4 μM (1.3 ± 0.4)	1.1 ± 0.5 g
+30 μM monepantel	23.5 μM (1.4 ± 0.7)	0.5 ± 0.4 g

In an effort to infer what nAChR subtypes monepantel maybe acting on, we generated inhibition plots for *Asu*-ACR-16, *Ode* pyrantel/tribendimidine and *Ode* levamisole subtype nAChRs, Fig. 5C and Table 5. The inhibition caused by monepantel (Fig. 5C) on *Asu*-ACR-16 had 2 components, one with an *IC*_*50*_ of 1.6 ± 3.1 nM, and the other with an *IC*_*50*_ of 0.2 ± 2.3 μM, suggesting the likelihood of more than one binding site for monepantel on *Asu*-ACR-16. The *IC*_*50*_ monepantel for *Ode* pyrantel/tribendimidine receptors (1.7 ± 0.7 μM) was seen to be lower than that for the *Ode* levamisole receptors (5.0 ± 0.5 μM). The *Ode* pyrantel/tribendimidine receptors appear to be more sensitive to monepantel than the *Ode* levamisole receptors. When we compare these results with that of the *in vivo* effects shown in Fig. 5B, monepantel appears to be acting on a mixture of the *Asu-*ACR-16, *Ode* pyrantel/tribendimidine and *Ode* levamisole nAChRs.

**Table 5:** *IC*_*50*_ values for monepantel on expressed nAChRs from *A. suum* and *O. dentatum*.

	*IC*_*50*_ (mean ± s.e.m., n)
*Asu*-ACR-16 nAChR	1.6 ± 3.1 nM; 0.2 ± 2.3 μM, n = 4
*Ode* pyrantel/tribendimidine nAChRs	1.7 ± 0.7 μM, n = 4
*Ode* levamisole nAChRs	5.0 ± 0.5 μM, n = 4

## 4 Discussion

### 4.1 Non-competitive antagonism of monepantel on expressed A. suum and O. dentatum receptors

In contrast to the positive allosteric modulatory effects of monepantel on DEG-3/DES-2 nAChRs, we found monepantel to produce non-competitive inhibition of *Asu-*ACR-16 and *Ode* levamisole sensitive and *Ode* pyrantel/tribendimidine sensitive nAChRs. These observations were consistent with results obtained from our muscle contraction assay. In all cases, monepantel produced no change in *EC*_*50*_ but a significant reduction in *R*_max_, an observation which was seen to be concentration-dependent. Of all three receptor subtypes expressed in *Xenopus* oocytes, *Asu*-ACR-16 was most sensitive to monepantel as reflected by its *IC*_*50*_ values of 1.6 ± 3.1 nM and 0.2 ± 2.3 μM. This was followed by the *Ode* pyrantel/tribendimidine receptor with an of *IC*_*50*_ of 1.7 ± 0.7 μM, and the *Ode* levamisole receptor with an *IC*_*50*_ of 5.0 ± 0.5 μM. Monepantel had a potent inhibitory effect on *Asu*-ACR-16, involving 2 components: one in which the maximum inhibition was only 40%, giving an *IC*_*50*_ value of 1.6 ± 3.1 nM and the other in which the maximum inhibition nearly reached 100%, giving an *IC*_*50*_ value of 0.2 ± 2.3 μM. These observations suggest that monepantel also acts via negative allosteric modulation, involving more than one binding site as is the case with abamectin and other negative allosteric modulators of nAChRs (Abongwa et al., 2016b, Zheng et al., 2016).

### 4.2 Mixed antagonism of monepantel on A. suum muscle flap

Monepantel causes hypercontraction of both *C. elegans* and *H. contortus* (Kaminsky et al., 2008a). In our electrophysiology experiments, monepantel produced an inhibitory effect on inward currents induced by acetylcholine. To further characterize the inhibition by monepantel, we tested different concentrations of monepantel in the presence of acetylcholine on *A. suum* muscle flaps. With all concentrations tested, monepantel produced a significant concentration-dependent reduction in *R*_max_, and a right-shift in *EC*_*50*_. In sharp contrast to the effects on *C. elegans* and *H. contortus* this indicates monepantel is a mixed antagonist of *Ascaris* muscle contraction. These results are likely due to the mixed nAChR populations on nematode muscle (Robertson et al., 1999, Robertson et al., 2002, Qian et al., 2006). *Asu-*ACR-16 is extremely sensitive to monepantel; the observed shift in *EC*_*50*_ in the muscle flap experiment is likely due to the almost complete inhibition of *Asu-*ACR-16 at the concentrations tested in the muscle. The non-competitive aspect of the inhibition is in agreement with the results obtained from the *Ode* levamisole sensitive and *Ode* pyrantel/tribendimidine sensitive receptors.

## 4.3 Conclusion

Our results indicate that monepantel acts as an antagonist of *Ascaris* muscle contraction, and as a non-competitive antagonist, with subtype selective effects, of expressed nAChR subtypes from *A. suum* (Clade III) and *O. dentatum* (Clade V). Non-competitive antagonism of monepantel on expressed nAChRs which we show in our research adds to the reported mode of action of monepantel as a positive allosteric modulator of expressed receptors of the DEG-3/DES-2 group of nAChRs. Thus, illustrating the complexity of the mode of action of the drug; involving more than one target site. Detailed understanding of the mode of action of antinematodal drugs is necessary, especially when considered for use in combination therapy/products, an approach proven to be highly effective for parasite control. As with many pharmacological agents we find that the mode of action of monepantel is complex and the drug is active on multiple nAChR subtypes.

## Statement of conflict of interest

None identified.

## Appendix A Supplementary data

Data Profile Data Profile
